# Erratum for “Peripheral and intestinal T lymphocyte subsets in dogs with chronic inflammatory enteropathy”

**DOI:** 10.1111/jvim.17262

**Published:** 2024-12-19

Agulla B, Villaescusa A, Sainz A, Díaz‐Regañón D, Rodríguez‐Franco F, Calleja‐Bueno L, Olmeda P, García‐Sancho M. Peripheral and intestinal T lymphocyte subsets in dogs with chronic inflammatory enteropathy. *J Vet Intern Med*. 2024; 38:1437. doi:10.1111/jvim.17036.

In the article cited above, there is an error in section 2.5 Statistical Analysis within section 2 Materials and Methods. The study used the Wilcoxon scores (rank‐sum) test—equivalent to the Mann‐Whitney U‐test—which is appropriate for comparing 2 independent groups. It was incorrectly referred to as the Wilcoxon's signed rank test, which is meant for paired data. This was a labeling mistake, not a methodological one.

We apologize for the error.

